# Evaluation of platelet indices as diagnostic biomarkers for colorectal cancer

**DOI:** 10.1038/s41598-018-29293-x

**Published:** 2018-08-07

**Authors:** Xianjin Zhu, Yingping Cao, Pingxia Lu, Yanli Kang, Zhen Lin, Taisen Hao, Yanfang Song

**Affiliations:** 1 Department of Laboratory Medicine, Fujian Medical University Union Hospital, 29 Xinquan Road, Fuzhou, 350001 China; 2 Department of Cancer Biology, Beckman Research Institute, City of Hope, Duarte, California, 91010 USA; 3 Department of Laboratory Medicine, Clinical Laboratory, Affiliated People’s Hospital of Fujian University of Traditional Chinese Medicine, 602 Bayiqi Road, Fuzhou, 350001 China

## Abstract

Altered platelet indices, including platelet count (PC), mean platelet volume (MPV), platelet distribution width (PDW), and plateletcrit (PCT), have been found in various cancer types. This study aimed to evaluate the role of platelet indices as potential biomarkers for the diagnosis of colorectal cancer (CRC), and to assess the association between platelet indices and CRC clinicopathological characteristics. The study included 783 subjects with CRC, 463 subjects with colorectal adenomas (CA), and 689 control subjects from June 2015 to October 2017. All participants’ clinicopathological characteristics were collected and analyzed. Here, we found that PC, MPV and PCT levels in CRC patients were significantly higher than those in CA patients and healthy participants (*p* < 0.001); however, PDW level in CRC patients was significantly higher than that in healthy participants while lower than that in CA patients. Receiver-operating characteristic (ROC) analysis indicated that combined detection of PCT and CEA appears to be a more effective marker to distinguish CRC patients from CA patients, with 70% sensitivity and 83% specificity. Among CRC patients, PC and PCT levels were associated with TNM stages and tumor size; MPV and PCT levels were associated with vascular invasion. Our findings suggest that altered PC, MPV and PCT levels might serve as potential biomarkers for the diagnosis and prognosis of CRC.

## Introduction

Colorectal cancer (CRC) is the third most common type of cancer worldwide, making up about 10% of all cases^1^. In recent years, the incidence and mortality of CRC has increased in China^2^. CRC-related death can be prevented through prompt screening. Currently, the CRC screening tests include colonoscopy, stool DNA tests, the fecal occult blood tests, and carcinoembryonic antigen (CEA) test or a combination assay of CEA and carbohydrate antigen 19-9 (CA19-9)^3,4^. Nevertheless, none of these tests is a reliable screening method due to their invasiveness, high cost, low sensitivity and specificity^5–8^. Therefore, it is urgent to identify simple, cost effective, and more sensitive biomarkers to improve the diagnosis and prognosis of CRC.

As we know, platelets play a vital role in the coagulation cascade. Recently, platelets were reported to be associated with the development and progression of malignancies as well^9–13^. New automated hematologic analyzers enabled to measure platelet count (PC) and platelets parameters with low cost. Parameters that are related to platelet are known as platelet indices, which mainly include platelet count (PC), mean platelet volume (MPV), platelet distribution width (PDW), and plateletcrit (PCT)^14–17^. MPV shows the average platelet volume in the blood, while PDW reflects the heterogeneity in platelet volume^18^. PCT serve as an indicator of the platelet mass in a unit of volume, and is calculated by PC and MPV^19^. Many studies have revealed the clinical significance of PC, MPV, PDW and PCT, indicating they might serve as potential markers for the diagnosis and prognosis of various cancer types, including lung cancer, breast cancer, gastric cancer, pancreatic cancer, laryngeal cancer and ovarian cancer^20–23^. However, previous studies showed conflicting data on the role of platelet indices as a diagnostic marker for CRC. Some studies reported that PC is increased in CRC patients and elevated PC is related to CRC patient survival^24–28^, while other studies failed to demonstrate the association between PC and the outcomes of CRC^29–33^. As for MPV and PDW, Li *et al*. found that MPV is elevated in colon cancer and this change is associated with tumor-nodule-metastases (TNM) stage of colon cancer^34^. Moreover, some studies suggested that MPV could be a valuable prognostic marker in CRC patients^35,36^. However, Wlodarczyk *et al*. reported that MPV in rectal cancer is significantly lower than that in healthy individuals^37^. Among the limitations of current research on the clinical significance of platelet indices in CRC are the following: First, the patient sample sizes of these studies are all less than 250, which might lead to a weaker power for the statistical significance. Second, there is no study for the clinical significance of PCT in CRC. Third, no paper to date clarified the diagnostic role of platelet indices in conjunction with other markers in patients with CRC, although it was widely recognized that biomarker combinations might have better diagnostic value than individual markers. Taken together, the clinical significance of PC, MPV, PDW and PCT in CRC still remains elusive.

In this study, we measured the levels of PC, MPV, PDW and PCT in a relatively large sample size, including patients with CRC, patients with colorectal adenomas (CA), and healthy controls. The diagnostic and prognostic value of platelet indices in CRC were examined in detail.

## Results

### The levels of PC, MPV, PDW and PCT in CRC patients

**Figure 1:**
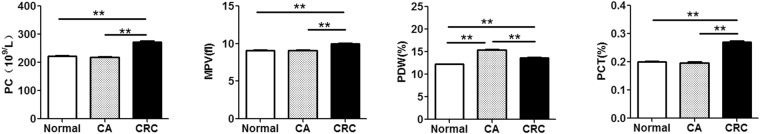
The levels of PC, MPV, PDW and PCT in CRC patients. The levels of PC, MPV, PDW and PCT were determined by hematology analyzer in CRC patients (n = 783), CA patients (n = 463), and healthy controls (n = 689). Data are presented as means ± SEM. **p < 0.01.

A total of 783 patients with CRC, 463 patients with colorectal adenomas (CA), and a control group of 689 healthy participants were enrolled in this study. PC, MPV, PDW and PCT levels in CRC patients, CA patients and healthy participants are shown in Fig. 1. The levels of PC, MPV and PCT were significantly higher in CRC patients than in CA patients and healthy participants; the level of PDW in CRC patients was significantly higher compared to healthy participants while lower than that in CA patients (Fig. 1). Our data demonstrated that altered PC, MPV, PDW and PCT levels are found in CRC patients compare to CA patients and healthy participants.

### Evaluation of PC, MPV and PCT as potential diagnostic biomarker for CRC

**Figure 2:**
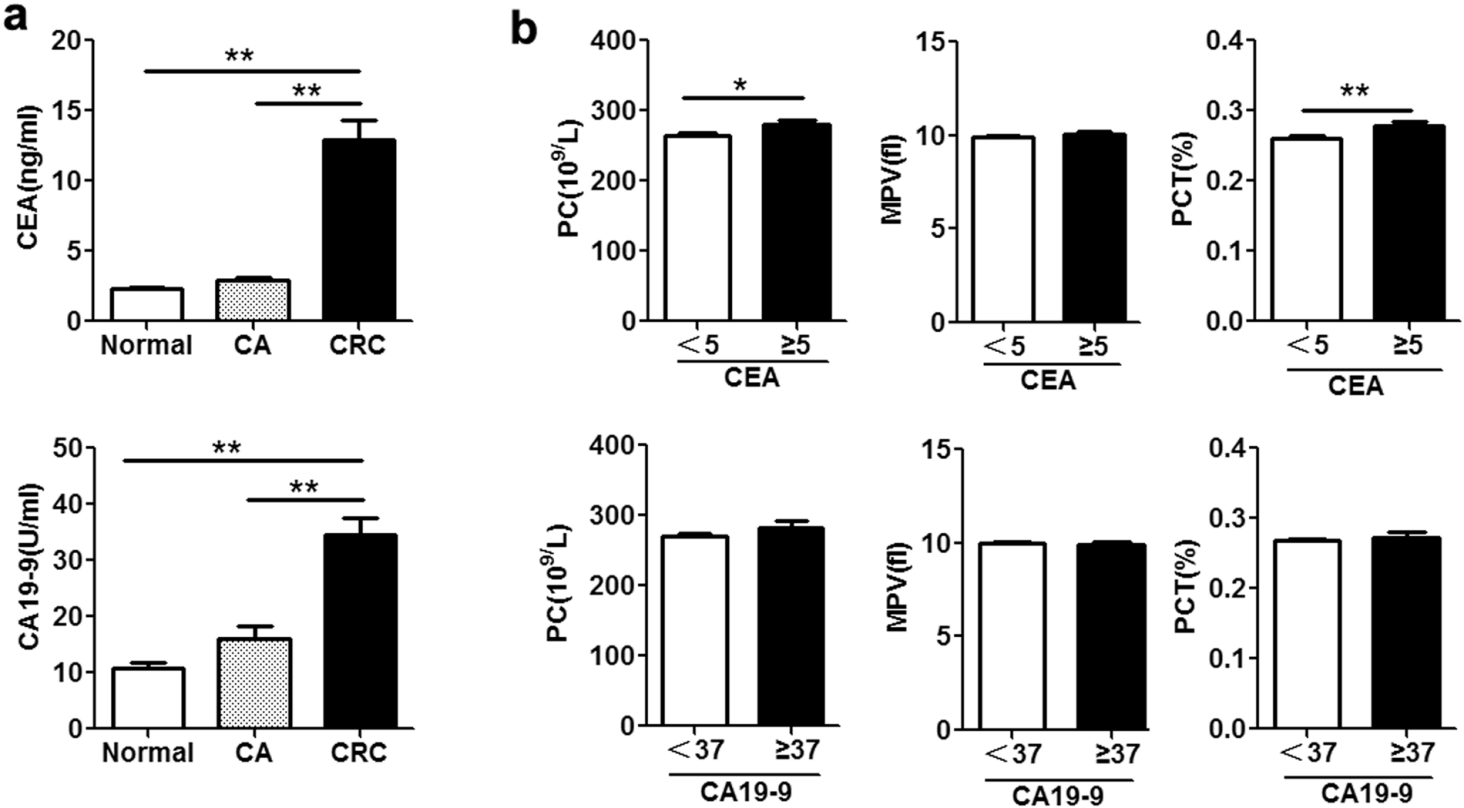
Relationship of PC, MPV, PDW, and PCT levels and CEA or CA19-9 levels in CRC patients. (**a**) Serum CEA (Up) and CA19-9 (Down) levels in CRC patients (n = 783), CA patients (n = 463), and healthy controls (n = 689) were measured by a Cobas 6000 Analyzer. (**b**) Up panel: The levels of PC, MPV and PCT in CRC patients with serum CEA levels of 5 ng/mL or more (n = 328) and below 5 ng/mL (n = 455) were analyzed by hematology analyzer. Down panel: The level of PC, MPV and PCT in CRC patients with CA19-9 levels of 37 U/mL or more (n = 132) and below 37 U/mL (n = 651) were analyzed by hematology analyzer. Data are presented as means ± SEM. *p < 0.05, **p < 0.01.

We assessed the role of PC, MPV and PCT as biomarkers for clinical diagnosis of CRC in comparison to CEA and CA19-9. Consistent with previous reports^38^, CEA and CA19-9 levels were significantly higher in CRC patients than in CA patients and healthy controls (Fig. 2a). Further analysis showed CRC patients with CEA level at least 5 ng/mL (the standard cut-off value) had elevated the levels of PC and PCT, but this phenomenon was not observed with MPV (Fig. 2b); In addition, PC, MPV and PCT levels showed no significant correlation with CA19-9 level of at least 37 U/mL (the standard cut-off value) (Fig. 2b).

**Figure 3:**
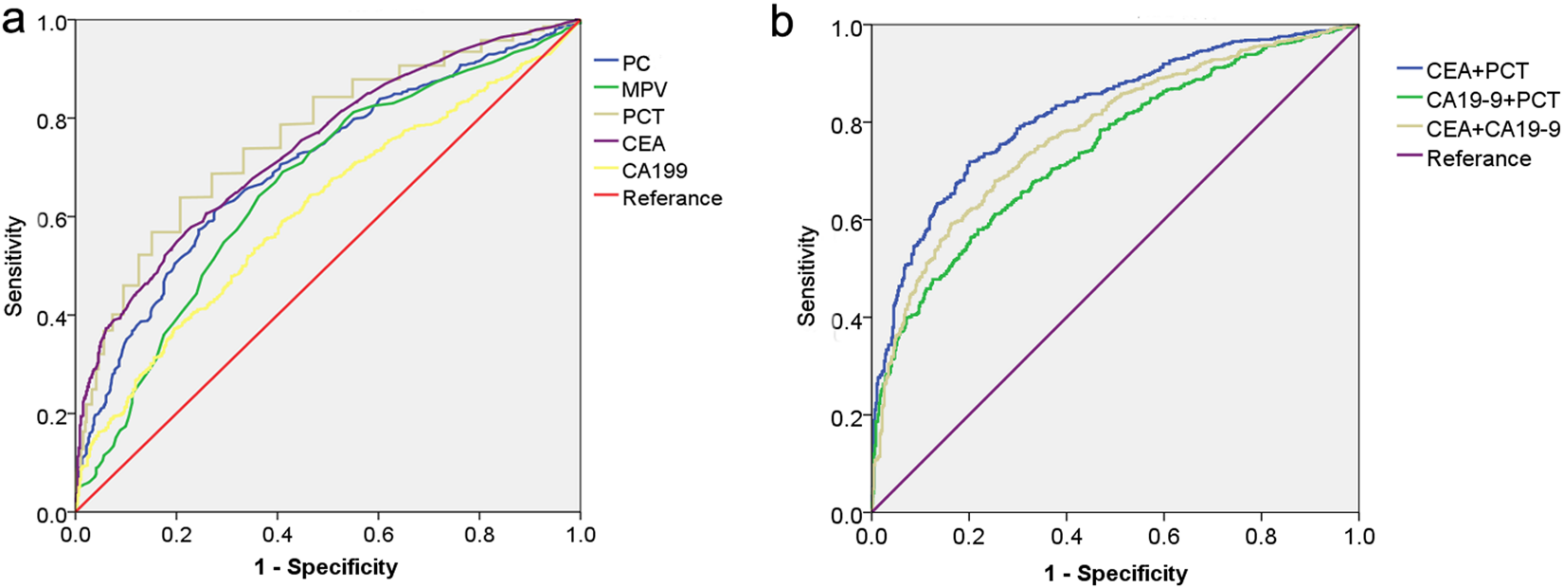
ROC analysis of the diagnostic performance of PC, MPV and PCT in comparison to CEA and CA19-9. (**a**) ROC curves of PC, MPV, PCT, CEA and CA19-9 alone for discriminating CRC patients from CA patients. (**b**) ROC curves of CEA + PCT, CA19-9 + PCT and CEA + CA19-9 for discriminating CRC patients from CA patients.

Next, we used a Receiver-operating characteristic (ROC) analysis to assess the availability of PC, MPV and PCT in the differential diagnosis of CRC patients and CA patients. The analysis showed the area under the ROC curve (AUC) for PC, MPV, PCT, CEA and CA19-9 were 0.706, 0.663, 0.765, 0.740 and 0.612, respectively (Table 1, Fig. 3a), indicating that PCT was the best one among these markers. When a PCT cut-off value of 0.230 was applied, we distinguished CRC patients from CA patients with a sensitivity of 64% and a specificity of 80% (Table 1). Using a CEA cutoff of 5 ng/mL led to a sensitivity of 41% and a specificity of 90%, and using a CEA cutoff of 37 U/mL yielded a sensitivity of 16% and a specificity of 94% in distinguishing CRC patients from CA patients.

**Table 1:** Diagnostic value of PC, MPV, PCT, CEA and CA199 alone and combined biomarkers for CRC.

variables	AUC	Cut off	sensitivity	specificity	95% confidence interval	
					upper limit	lower limit
PC	0.706	242.50	62%	72%	0.677	0.735
MPV	0.663	9.25	69%	59%	0.632	0.694
PCT	0.765	0.230	64%	80%	0.738	0.791
CEA	0.740	5.00	41%	90%	0.713	0.767
CA19-9	0.612	37.00	16%	94%	0.580	0.643
CEA + PCT	0.835		70%	83%	0.812	0.857
CA19-9 + PCT	0.781		61%	83%	0.756	0.807
CEA + CA19-9	0.743		58%	78%	0.716	0.770

Then, PCT in conjunction with CEA or CA19-9 were further analyzed for combined detection. As shown in Table 1 and Fig. 3b, the AUC of the combined detection of PCT and CEA was significantly higher than the AUC of CEA alone, the combined detection of CEA and CA19-9, and the combined detection of PCT and CA19-9. Importantly, when PCT and CEA were combined, sensitivity and specificity are 70% and 83%, respectively.

Taken together, these results suggested that combined detection of PCT and CEA performs better than the detection of CEA alone, combined detection of CA19-9 and PCT, and combined detection of CEA and CA19-9 in discriminating CRC patients from CA patients, with higher sensitivity and specificity.

### The relationship between platelet indices and clinicopathological characteristics in CRC patients

As shown in Table 2, the level of PC was significantly greater in patients with more advanced TNM stages (*p* < 0.0001). Further analysis showed that the level of PC was significantly associated with age, location, primary tumor stages (pT stages) and tumor size. However, no significant correlation was observed between PC level and gender, lymph node stages (pN stages), distant metastasis stages (pM stages), vascular invasion and differentiation (Table 2). As for MPV, there was significant correlation between MPV and vascular invasion (Table 2). PCT was associated with the tumor location, TNM stages, pT stages, pN stages, vascular invasion, and tumor size. However, none of the clinicopathological features was significantly associated with the PDW. Taken together, these data indicated that PC and MPV and PCT correlate with clinicopathological characteristics in patients with CRC.

**Table 2:** Relationship between platelet indices and pathological characteristics in CRC patients.

Variables	N	PC (×10^9^/L)	*p*	MPV (fl)	*p*	PDW (%)	*p*	PCT (%)	*p*
Gender			*0*.*1698*		*0*.*6367*		*0*.*4163*		*0*.*8311*
Male	467	269.2 ± 4.051		10.17 ± 0.2230		13.54 ± 0.1374		0.271 ± 0.0070	
Female	316	277.2 ± 4.799		10.37 ± 0.3965		13.73 ± 0.1959		0.269 ± 0.0049	
Age			***0***.***009***		*0*.*9548*		*0*.*6974*		*0*.*4726*
≤60	357	281.5 ± 4.665		10.27 ± 0.3542		13.67 ± 0.1383		0.274 ± 0.0047	
>60	426	265.3 ± 4.110		10.24 ± 0.2405		13.58 ± 0.1743		0.267 ± 0.0075	
Location			***0***.***0002***		*0*.*3219*		*0*.*4754*		***0***.***002***
Colon	430	283.2 ± 4.691		10.22 ± 0.2400		13.54 ± 0.1639		0.279 ± 0.0049	
Rectum	353	259.9 ± 3.698		9.950 ± 0.0871		13.71 ± 0.1548		0.258 ± 0.0043	
TNM stage			<***0***.***0001***		*0*.*6727*		*0*.*7132*		***0***.***0002***
I	136	238.9 ± 5.612		9.981 ± 0.1280		13.81 ± 0.2208		0.237 ± 0.0065	
II	247	275.5 ± 6.178		9.857 ± 0.1244		13.71 ± 0.2402		0.266 ± 0.0059	
III	322	278.5 ± 4.884		10.07 ± 0.1329		13.47 ± 0.1696		0.278 ± 0.0056	
IV	78	284.0 ± 8.675		10.05 ± 0.2261		13.60 ± 0.3025		0.282 ± 0.0097	
pT stage			<***0***.***0001***		*0*.*7673*		*0*.*2589*		<***0***.***0001***
T1	51	240.2 ± 8.221		9.780 ± 0.1591		13.67 ± 0.3514		0.232 ± 0.0075	
T2	120	240.2 ± 5.984		9.978 ± 0.1385		13.76 ± 0.2358		0.239 ± 0.0072	
T3	481	274.5 ± 3.982		10.21 ± 0.2162		13.66 ± 0.1586		0.271 ± 0.0043	
T4	131	299.6 ± 8.980		9.928 ± 0.1603		13.29 ± 0.2429		0.292 ± 0.0082	
pN stage			*0*.*0814*		*0*.*2524*		*0*.*122*		***0***.***034***
N0	393	267.2 ± 4.511		9.916 ± 0.0931		13.72 ± 0.1711		0.261 ± 0.0045	
N1	240	279.3 ± 5.094		10.17 ± 0.1722		13.74 ± 0.1993		0.282 ± 0.0066	
N2	150	280.4 ± 6.385		9.873 ± 0.1217		13.14 ± 0.2224		0.270 ± 0.0075	
pM stage			*0*.*2541*		*0*.*7885*		*0*.*6413*		*0*.*2389*
M0	707	271.5 ± 3.296		9.979 ± 0.0784		13.60 ± 0.1220		0.268 ± 0.0036	
M1	76	283.5 ± 8.831		10.05 ± 0.2335		13.78 ± 0.2982		0.282 ± 0.0099	
Differentiation			*0*.*7167*		*0*.*2818*		*0*.*818*		*0*.*818*
Poor	80	276.5 ± 10.31		9.703 ± 0.2728		13.96 ± 0.4597		13.96 ± 0.4597	
Moderate	637	271.3 ± 3.393		9.962 ± 0.0781		13.62 ± 0.1308		13.62 ± 0.1308	
Well	65	256.7 ± 20.12		9.325 ± 0.2683		13.86 ± 0.6398		13.86 ± 0.6398	
Invasion			*0*.*7875*		***0***.***0119***		*0*.*1188*		***0***.***0417***
Negative	426	272.4 ± 4.390		9.780 ± 0.0833		13.61 ± 0.1622		0.265 ± 0.0044	
Positive	257	274.3 ± 5.409		10.79 ± 0.4972		13.21 ± 0.1907		0.280 ± 0.0063	
Tumor size (cm)			<***0***.***0001***		*0*.*8444*		*0*.*3427*		***0***.***0002***
<5	368	256.2 ± 4.571		9.871 ± 0.0923		13.92 ± 0.1549		0.250 ± 0.0047	
≥5	415	297.8 ± 7.246		9.829 ± 0.2160		14.23 ± 0.3032		0.277 ± 0.0044	

Values of PC, MPV, PDW and PCT are expressed as mean ± standard error. Bold indicates a statistically significant.

## Discussion

In this study, we are the first to find that PCT plays an important role in the diagnosis of CRC, and the combined detection of PCT and CEA can achieve better diagnostic performance than combined detection of CEA and CA19-9 in discriminating CRC patients from CA patients, with higher sensitivity and specificity. Moreover, among CRC patients, PC and PCT levels are associated with TNM stages and tumor size; MPV and PCT levels are associated with vascular invasion. These results indicated that PC, MPV and PCT might function as potential diagnostic and prognostic markers for CRC.

Previous studies have suggested that platelets play a significant role in cancer progression and metastasis^11–13^. The relatively low cost, high reproducibility and high applicability of laboratory measurement for PC, MPV, PDW and PCT make them suitable for clinical usability. Growing numbers of evidence suggested that PC, MPV, PDW and PCT can be potentially developed as markers for the diagnosis and prognosis of various cancers^20–23^.

The colorectal adenoma (CA), a precursor lesion of CRC, is a benign glandular tumor of the colon and the rectum. The majority of CRC arise from transformation of CA, but only 5% of CA progress to cancer. It is difficult to discriminate CRC patients from CA patients, unless the colonoscopy screening is adopted^39^. Colonoscopy screening is not a reliable screening method due to their invasiveness of the procedure, high cost, inappropriate perception of risk, dietary restrictions. Previous studies have suggested that PC, MPV, PDW and PCT have a potential role for the diagnosis of various cancers^20–23^, whether PC, MPV, PDW and PCT can serve as markers for discriminating CRC patients from CA patients remains unknown.

First, we found that PC, MPV, PCT and PDW levels were significantly higher in CRC patients compared to CA patients and healthy participants. The level of PDW in CRC patients was significantly higher compared to healthy participants while lower than that in CA patients. Then, we evaluated the performance of PC, MPV and PCT as the diagnostic marker of CRC by ROC analysis, and the results showed that PCT can discriminate CRC patients from CA patients with better performance than CEA and CA19-9, which are widely used as markers for the diagnosis of CRC^40^. It was widely recognized that marker combinations have better diagnostic performance than individual markers. PCT in conjunction with CEA or CA19-9 were further analyzed. We found that combined detection of PCT and CEA discriminates CRC patients from CA patients with higher sensitivity and specificity.

Next, we assessed the association between platelet indices and clinicopathological characteristics of CRC patients. Previous studies found that increased PC is correlated with poorer prognosis in various types of cancer^41–44^. Our results showed that increased PC in CRC patients was not only associated with the TNM stages but also showed strong correlation with tumor size. MPV shows the average platelet volume in the blood, to our knowledge, we report here for the first time that MPV was associated with vascular invasion, but our results failed to demonstrate the association between MPV and the tumor differentiation of CRC^45^. PCT, a factor that is calculated by PC and MPV, is now generally recognized to reflect platelet activity and the percentage of platelets in blood^19^. To date, PCT is only found to be higher in epithelial ovarian cancer, papillary thyroid carcinoma^14^, and pancreatic adenocarcinoma^46^. Our results firstly showed that PCT was increased in CRC patients. Moreover, increased PCT level was associated with TNM stages, vascular invasion, and tumor size. PDW is a coefficient of variation of the platelet volume average, which was only described by one study reporting that elevated PDW is significantly associated with TNM stage and predicted worse prognosis in non-metastatic CRC patients^36^. However, our results showed that none of the clinicopathological features was associated with the PDW in CRC patients. Taken together, these findings showed that PC and PCT levels are associated with TNM stages and tumor size, and MPV and PCT levels are associated with vascular invasion in CRC patients. Considering that TNM stage, tumor size and vascular invasion are poor prognostic factors for CRC patients^47,48^, PC, MPV and PCT should be employed as potential markers for the prognosis of CRC.

There are several possible explanations for our findings. On the one hand, tumor cells can produce factors leading to the proliferation and differentiation of megakaryocyte, thereby promoting platelet production and activation^11^. On the other hand, activated platelets not only can protect tumor cells against cytolysis, but also release some growth factors contributing to cancer cell growth, metastases and invasion^11,13,49–52^. These processes initiate a cross-talk between platelets and tumor, which promotes tumor growth and invasion, and platelet production and activity.

This study is limited in the following aspects. First, the short follow-up duration of the study with single-centered retrospective design might raise bias towards sample selection and analysis. Second, we failed to collect data on overall survival, thus we didn’t evaluate the relationship of platelet indices with overall survival in CRC patients. Third, racial diversity can be a confounding factor since only Chinese ethnicity was included in the study. Therefore, more follow up studies on populations with higher racial, geological and national diversity in separate laboratories should be carried out to confirm the results herein.

In summary, despite these limitations, our study is the first study that systematically evaluated the role of PC, MPV, PDW and PCT in CRC in a relatively large sample size. Our results indicated that combined detection of PCT and CEA should be considered as potential effective markers for the clinical diagnosis of CRC. Also, PC, MPV and PCT levels can be potentially utilized as biomarkers for the prognosis of CRC. However, our data is still preliminary and the clinical application of platelet indices requires further investigation by additional clinical studies in a larger and diversified population.

## Materials and Methods

### Patients’ characteristics

Samples from 783 patients (467 men and 316 women, age range: 21–92 years, mean ± SD age: 60.66 ± 12.05 years) with the newly diagnosis of CRC without receiving any treatment were included in this study. The controls were 463 patients with colorectal adenomas (312 men and 151 women, age range: 21–88 years, mean ± SD age: 59.05 ± 11.25 years) and 689 healthy age-matched volunteers (383 men and 316 women, age range: 23–87 years, mean ± SD age: 59.82 ± 12.77 years). All CRC patients were diagnosed at Fujian Medical University Union Hospital (Fuzhou, China) from June 2015 to October 2017. The clinicopathological data of all CRC patients were collected, including age, sex, tumor size, tumor location, tumor differentiation, TNM stage, and vascular invasion. Patients were excluded if they had hematological disorders, coronary artery disease, hypertension, diabetes mellitus, and medical treatment with anticoagulant, and acetylic salicylic acid. This study was performed in accordance with ethical guidelines and was approved by the Institutional Medical Ethics Review Board of Fujian Medical University Union Hospital. Informed consent was obtained from all included participants.

### Analysis of PC, MPV, PDW and PCT levels

Peripheral blood samples were obtained from newly diagnosed CRC patients. PC, MPV, PDW and PCT were measured routinely with Beckman Coulter LH 780 hematology analyzer (Beckman Coulter, Brea, CA, USA) according to the manufacturer’s instructions.

### Analysis of serum CEA and CA19-9 levels

Peripheral venous blood was obtained from newly diagnosed without receiving any treatment CRC patients. The serum levels of CEA and CA19-9 were determined by a Cobas 6000 Analyzer (Roche Diagnostics, Mannheim, Germany). According to the manufacturer’s instructions, the cut-off value for normal CEA is less than 5 ng/mL, and that for normal CA19-9 is less than 37 U/mL.

### Statistical analysis

Statistical analysis was performed using SPSS version 21.0 software or GraphPad Prism version 5.0. All data were expressed as means ± standard error of mean (SEM). The levels of PC, MPV, PDW and PCT of CRC, CA and healthy controls were analyzed using unpaired Student’s t test. Receiver-operating characteristic (ROC) curves were used to evaluate the diagnostic role of PC, MPV and PCT in distinguishing CRC patients from CA patients. The diagnostic accuracy of the ROC curve was determined by area under the curve (AUC), and the AUC value closer to 1 indicates that the diagnostic test is perfect. The Youden index was used to determine the optimal cut-off value for PC, MPV and PCT to differentiate between CRC patients and CA patients^53^. Differences in PC, MPV, PDW and PCT as grouped by clinicopathological characteristics were compared by an unpaired Student’s t test or ANOVA with Student-Newman-Keuls tests. All tests were two-tailed and a threshold of *P* < *0*.*05* was defined as statistically significant.
